# Economic methods and spatial scales in One Health: Results from a scoping review

**DOI:** 10.1016/j.onehlt.2025.101115

**Published:** 2025-06-21

**Authors:** Lena Schmeyers, Susan Thomschke, Lena Victoria Mende, Greet Stichel, Daniel Schiller, Steffen Fleßa

**Affiliations:** a University of Greifswald, Faculty of Law and Economics, Chair of General Business Administration and Health Care Management, Friedrich-Loeffler-Straße 70, 17489 Greifswald, Germany; b University of Greifswald, Faculty of Mathematics and Natural Sciences, Institute of Geography and Geology, Chair of Economic and Social Geography, Friedrich-Ludwig-Jahnstraße 16/17a, 17489 Greifswald, Germany

**Keywords:** One Health, Region, Geography, Economics, Costs, Healthcare

## Abstract

**Introduction:**

COVID-19 and other zoonoses indicate the close connection between human, animal, and environmental health. This interdependency underscores the need for a comprehensive One Health approach. However, the One Health concept is sometimes reduced to combating zoonoses and antimicrobial resistance, neglecting the spatial and environmental dimensions. This narrow focus overlooks the potential of One Health in geographic contexts, where it can optimize health within regional ecosystems. Therefore, this paper aims to provide an overview of geographic contexts and economic approaches to measuring One Health and the importance of these factors for effective health outcomes.

**Material and methods:**

A comprehensive search for economic evidence and the geographical scope of One Health was conducted. The search terms ‘One Health’, combined with ‘region, landscape, area, geography, cost, economics, utility,’ were used in Web of Science, Scopus, and PubMed. Articles were screened by two blinded reviewers. Year, author, economic method, intervention, outcome, study aim, topic, and geographical area of the articles were recorded.

**Results:**

1214 articles were retrieved and 108 were included in this analysis. The topics focused on: zoonoses (56 %), antimicrobial resistance (14 %), food safety/security (7 %), animal welfare (6 %), and governance (6 %). Most studies were conducted in African countries, the majority of studies (57 %) adopted a regional perspective, while 19 % employed a national and 13 % adopted a multi-country perspective. The most common economic approaches were mixed methods and CEA, regression analysis, as well as index methods.

**Discussion:**

The analyzed articles largely focus on zoonoses and current measurement instruments that do not yet align with the requirements of the One Health Joint Plan of Action 2022–26. Integrating geographical considerations promises a more comprehensive and effective approach to One Health challenges. The diversity of identified measurement instruments provides a valuable foundation for developing future, context-sensitive One Health strategies.

## Highlights

• Most used economic methods for measuring One Health were mixed methods, index methods, Cost-Effectiveness and regression analysis.
• The majority of articles focused on the topic of zoonoses and Antimicrobial Resistance.
• There is a lack of consideration of the One Health concept in regional contexts.
• Most studies were conducted in African countries.
• The geographical context most often referred to were regional scales at a rural area level.

## 1 Introduction

The impact of the SARS-CoV-2 pandemic was felt not only during the active phase, but also in the years that followed. More than 7 million people have already died from coronavirus disease. The high number of hospitalizations and the loss of trillions of US dollars worldwide illustrate the significant impact of the pandemic on health systems and populations.[1,2] Pandemics such as SARS-CoV-2 demonstrate the importance of joint efforts, particularly in regions with limited health infrastructure [3,4]. In view of the climate crisis and the changing habitat of humans, animals and the environment, holistic health strategies have never been as relevant as they are today [5]. It is therefore necessary to develop and implement long-term and sustainable health strategies that optimize the use of available human and financial resources.

In the 20th century, the concept of One Medicine was a groundbreaking innovation for the understanding of health as an interdependence between human and animal health and it was at this time the most efficient health strategy [6,7]. Nowadays we know that considering animal and human health separately from environmental health is insufficient. One Medicine developed into a more comprehensive, holistic health strategy: One Health. One Health is defined by the One Health High-Level Expert Panel as “(…) an integrated, unifying approach that aims to effectively balance and optimize the health of people, animals, and ecosystems. (…) The concept mobilizes multiple sectors, disciplines, and communities at varying levels of society to work together to foster well-being and tackle threats to health and ecosystems, while addressing the collective need for healthy food, water, energy, and air, taking action on climate change and contributing to sustainable development.”[8] To achieve the implementation of One Health on a global scale, the One Health Joint Plan of Action for the period 2022–2026 has been developed by the Quadripartite Organizations, namely the Food and Agriculture Organization, the United Nations Environment Programme, the World Organization for Animal Health, and the World Health Organization. The plan outlines the procedures and timeframes for enhancing One Health's capacities. These capacities pertain to the following areas: food safety, the reduction of antimicrobial resistance risks (e.g., the loss of bacterial responsiveness to antimicrobial medication), the control of zoonoses, neglected tropical, vector-borne diseases, and the inclusion of the environment [9].

It has already been proven that the One Health concept can contribute to cost reduction, for example, through interdisciplinary cooperation, in the prevention of zoonoses, infectious diseases, and pandemics, as well as through research and development [10,11]. Some countries have already implemented national One Health strategies, such as the USA, Tanzania, India, and Thailand, in contrast to Germany, Poland and China [12]. The implementation may encounter several challenges, including a lack of evidence concerning their monetary value, insufficient human resources, weak coordination and collaboration between different sectors, the need for sustainability strategies after the end of project funding, and the influence of local political and economic dynamics [13,14]. Although a cost reduction has been proven, One Health is still frequently used selectively for certain topics, i.e., the main thematic focus of One Health strategies lies on preventing zoonoses and antimicrobial resistance [15]. Likewise, 80.8 % of funding is used for the topic of zoonoses in the One Health area [16]. Other topics such as ‘Environmental and Ecological Issues’ and ‘Sustainable Food Systems’ have thus far been neglected, as a comparison of abstracts from the World One Health Congress from 2011 and 2020 demonstrated. Furthermore, the discrepancy regarding the implementation of One Health between countries with a high and low human development index was highlighted [17].

Although numerous One Health approaches exist for specific diseases, there is currently no comprehensive overview of the different economic measurement methods and geographical contexts. Regional differences in terms of ecosystems and environmental factors as well as levels of economic development and configurations of health systems are expected to result in region-specific economic outcomes of One Health approaches. Regions are areas that are characterized by certain features, e.g., climate zone, government boundaries or economic conditions. A region can refer to a multi-national level, including several countries, such as the African Region or it refers to sub-national units below national boundaries, such as Provence-Alpes-Côte d'Azur in southeastern France [18]. In this article, the terms “region” and “regional” are used to refer to sub-national contexts and local areas.

The utilization of One Health metrics, values and returns is imperative to enhance public health efficiency and to implement this comprehensive approach in the long term [11,19]. The utilization of economic measurements has the potential to guide decisions in managing limited resources, thereby ensuring optimal outcomes. Furthermore, standardized measurements can be employed to plan additional One Health approaches and build evidence for changing policies. To gain insight into the economic measurements that have been used in the regional implementation of One Health, we conducted a scoping review. This review provides an overview of the figures, geographical contexts, and thematic distribution that have been commonly used. Further, this study addresses the following research questions:

1. What economic evidence is available for the regional context of One Health?
2. What thematic foci exist in this field of One Health in a geographical context?

## 2 Methods

### 2.1 Overview

**Fig. 1:**
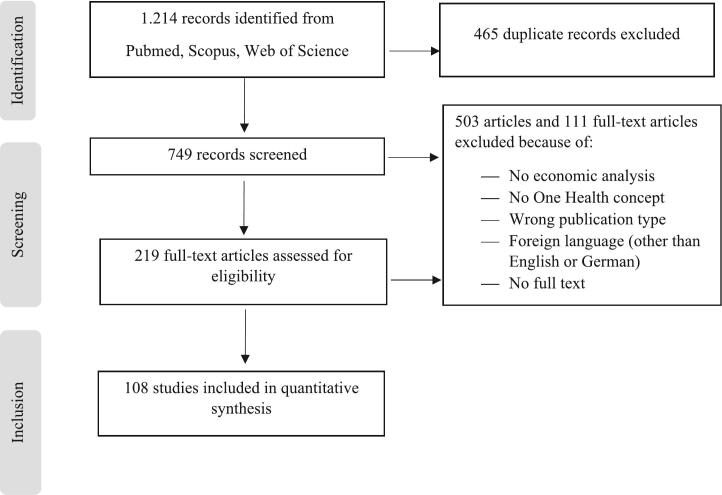
Scheme for the literature search process.

The objective of this scoping review was to identify and analyze knowledge gaps [20] following the PRISMA statement [21], as illustrated in Fig. 1.

### 2.2 Identification

A literature search was performed in July 2024 in the databases Web of Science and Scopus, since they are the databases with the highest number of scientific literature, see Fig. 1. With the search conducted in Pubmed, more studies from the field of health economics in particular, were thought to be found. The following search string was used: (“One Health” AND (space* OR region* OR landscape* OR area* OR place* OR geograph*) AND (cost* OR econom* OR util*)).

**Fig. 2:**
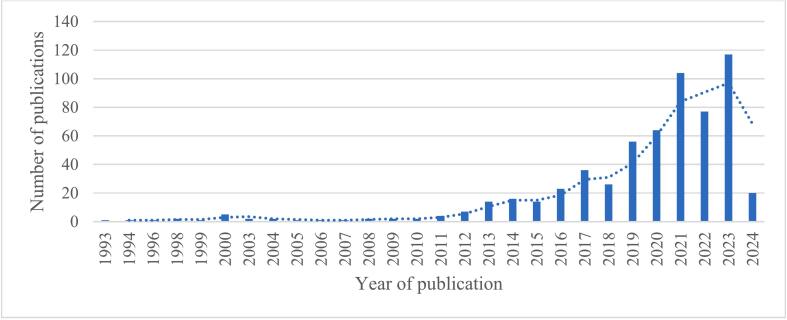
Number of publications retrieved in Web of Science.

The search of the Web of Science and Scopus databases revealed that the majority of articles were published within the last 10 years, see Fig. 2. Consequently, an additional search of the PubMed database was conducted within this time frame.

Only articles in English and German were considered. Based on these search terms, 1.214 original articles were retrieved in total.

### 2.3 Screening

Following the removal of duplicates, 749 records were screened in a blinded manner by at least two individuals utilizing a review management software, Rayyan (see https://www.rayyan.ai/), which enables group access. In the event of a discrepancy in the ratings of an article (e.g., Reviewer 1: excluded and Reviewer 2: included), it was discussed and re-evaluated. This process led to the clarification of the inclusion criteria.

### 2.4 Inclusion

An article was identified as a relevant One Health article for this review when two areas of the three domains (health of animals/human/environment) were considered and the term “One Health” was mentioned in the text. Studies were included if they used an economic method to implement One Health in a geographical context. For the geographical context, the particular scale and area were considered. Scale was defined as global, multi-country, national, and regional (sub-national). An area was defined as rural, urban and/or peri-urban, urban and rural [18,22].

The economic methods considered for inclusion consisted of several approaches. Cost-Effectiveness-Analysis (CEA) was considered, where costs are measured in monetary units and benefits are measured in natural units, such as reduction in blood pressure (measured in mm Hg) [23]. Cost estimation approaches, which focus on quantifying the expenditure required for interventions or measures without necessarily comparing alternatives or assessing benefits, were also included. For instance, direct costs such as medication or labor time can be used. [24] Interventions aimed at cost-effectiveness (CE) indicate that a desired outcome should be achieved with the lowest possible resource use [25]. Another approach was the Cost-utility Analysis (CUA), where costs are measured in monetary units and benefits are measured in disease-related or health-related units that consider quality of life [23]. The concept of burden of disease includes direct costs (e.g., treatment, hospitalization), indirect costs (e.g., productivity loss), and intangible costs (e.g., quality of life), often measured using Disability-Adjusted Life Years (DALYs) or Quality-Adjusted Life Years (QALYs). The impact on quality of life can be measured in QALYs, which measure the gain in quality and duration of life, while DALYs quantify the loss of healthy life years due to illness and death. [[26], [27], [28]] A Cost-Benefit Analysis (CBA) measures both costs and benefits in monetary units, for instance, by using willingness-to-pay to calculate health effects, which represents how much a person would pay to receive a health benefit. The Cost of Illness (COI) is a summary of the costs incurred by society as a result of a particular illness, such as treatment and loss of productivity [29]. Another analysis, the Cost-Minimization Analysis (CMA), identifies the least costly option among alternatives with identical effectiveness and safety profiles [26]. For the modeling of processes, Markov models were included for sequential decision-making processes [30] and Monte Carlo simulation for simulations of random-based scenarios [31]. Other weighting factors, such as the quantified valuation by scoring, weighting, and ranking, were incorporated as well. Furthermore, statistical analyses were included, such as logistic regression analysis, which are used for measuring the influence of variables on binary outcomes (e.g., whether the patient is ill or not) in contrast to multiple linear regression analysis with numeric outcomes (e.g., days till death) [32]. Qualitative analyses were also included, which can be used to depict more complex contextual factors that cannot be represented in economic models. Health economic analyses require the consideration of different stakeholder perspectives (patients, service providers, payers), which can be systematically captured through qualitative research [33].

Thematic categories were initially established based on a preliminary review of the literature on PubMed [34]. The categories were as follows: AMR (Antimicrobial resistance), animal welfare, environment, food safety, governance, veterinarians, and zoonoses. For each article, the year, author, economic method, intervention, outcome, study aim, and geographical context were recorded.

### 2.5 Exclusion

Publication types, such as reviews, background articles, and commentaries, were excluded, as well as articles without full-text access. Articles for which full-text access was not available due to restrictions beyond the institutional license were excluded from the review. This criterion was necessary to enable a thorough and reliable assessment of each study's economic methodology and geographic context. Articles that were not in English or German were also removed. Articles mentioning One Health, but not meaning the One Health concept, such as a one health state, a one health worker, a one health measure, a one health center, a number one health problem, a one health education, a one health visit, were not considered. Studies considering only one aspect of human/animal/environmental health were excluded. Articles with no economic consideration, but for example with a focus on microbial analysis or reporting implementation problems, were eliminated. A total of 503 articles were excluded at the screening stage, based on the abstract, title, and/or keywords. Further 111 articles were excluded at the full-text review stage, as they did not meet the pre-established inclusion criteria.

## 3 Results

### 3.1 Geographical context of One Health research

**Fig. 3:**
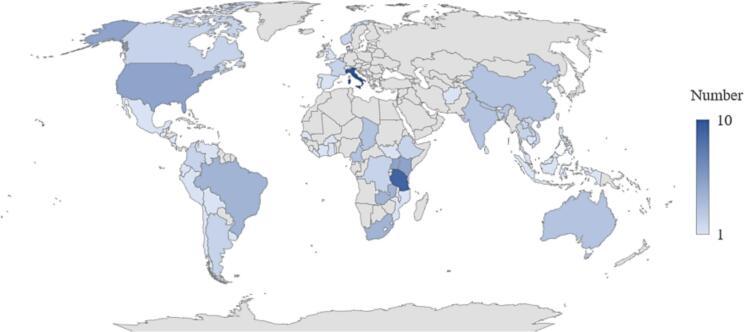
Global map showing the number of articles on the topic of economic analysis of One Health with territorial reference.

Most of the 108 analyzed studies were conducted in African countries (*n* = 49), of which 9 involved Tanzania [[35], [36], [37], [38], [39], [40], [41], [42], [43]], 5 Kenya [[44], [45], [46], [47], [48]] and 5 Uganda [[49], [50], [51], [52], [53]], see Fig. 3. The second most frequently included countries were located in Europe (*n* = 27), with Italy being the most common one (*n* = 10) [[54], [55], [56], [57], [58], [59], [60], [61], [62], [63]]. Asian countries were the third most current category (*n* = 20), with a small number of studies being carried out in several Asian countries, e.g., 3 in India [[64], [65], [66]], 3 in China [[67], [68], [69]], 3 in Nepal [[70], [71], [72]].

A more detailed examination of the geographical scale reveals that 62 of the 108 included papers adopted a regional perspective (57 %), focusing on specific areas in a single country. Subsequently, 20 papers (19 %) adopted a national research perspective, while 14 papers (13 %) employed a multi-country perspective. A total of nine papers (9 %) focused on a global scale, while three (3 %) did not consider any geographical scale, as they pertained to the examination of mathematical models. Notwithstanding their economic characteristics, they were nevertheless included in the analysis.

In the majority of papers, no further small-scale analysis could be identified (*n* = 49; 45 %). In the papers with a further differentiation of research areas, the focus is mainly on rural areas (*n* = 30; 27 %). This is followed by an exclusively urban and/or peri-urban research area perspective (*n* = 15; 14 %) and an area level that considered both urban and rural areas (*n* = 14; 13 %). It is important to note that the area levels vary quite considerably across the various scales. As demonstrated in Table 1, studies undertaken at regional levels have been found to prioritize the focus on rural areas.

**Table 1:** Geographical contexts regarding One Health approaches: Scale and area considerations.

		Scale					
		Global	Multi-country	National	Regional	No scale consideration/ unknown	Total
Area	Rural	0	2	1	27	0	30
	Urban and/or peri-urban	0	0	2	13	0	15
	Urban and rural	0	0	1	13	0	14
	No urban/rural consideration/ unknown	9	12	16	9	3	49
	Total	9	14	20	62	3	108

### 3.2 Economic approaches and geographical context for articles focusing on zoonoses

**Fig. 4:**
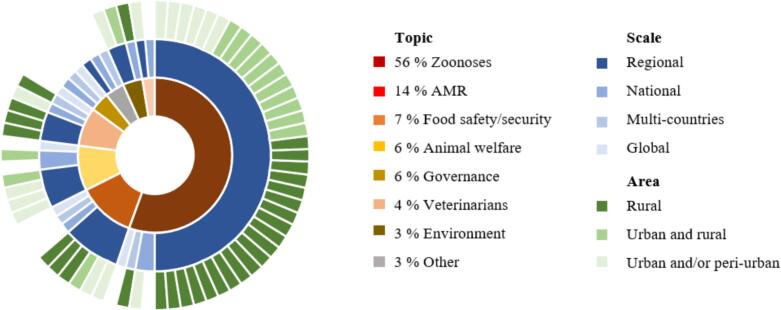
Thematic focus and geographical context of One Health articles.

The majority of papers focused on zoonoses (*n* = 61; 56 %), see Fig. 4, mainly on rabies (*n* = 19; 31 %), followed by general zoonoses prevention (*n* = 8; 13 %) and less than 5 % each tackled Anthrax [39,39], Avian Influenza [63], Brucellosis [46,69], Echinococcosis [59,61,73], infectious diseases [36,74], intestinal Parasitosis [75], Leishmaniasis [61], Leptospirosis [76,77], Neurocysticercosis [52], catarrhal Fever [41], Opisthorchiasis [77], Peste de petit [53], Rift Valley fever [44,78], Schistosomiasis [79], Sleeping sickness [49,51], Snakebites [[70], [71], [72]], Taenia solium [52,80,81], tick-borne diseases [50,58,82], Toxoplasma gondii [83], Triatomines [84], Tuberculosis [85] and West Nile Virus [60,62], see Table 2.

**Table 2:** Economic One Health approaches for zoonoses.

Author	Year	Source	Economic approach	Intervention	Outcome	Study aim
Defilippo et al.	2022	[60]	CEA	Integrated West Nile surveillance program	Costs for environmental and veterinary surveillance, blood transfusion, infections in humans	CE of surveillance against West Nile Virus in humans
Fasina et al.	2020	[43]	CEA, Burden of disease	Protective vaccination ring around a city at risk	Incidence, YLG (DALYs estimated), CE ratio, disease mapping, 2 scenarios vaccination coverage, implementation time	CE of rabies vaccination for humans
Bilinski et al.	2022	[40]	CEA, Burden of disease, Rabies virus transmission model	Mass dog vaccination campaigns, surveys, interviews, observations	Vaccination costs, purchasing power parity, DALYs, mortality	CE of rabies vaccination for animals
Léchenne et al.	2016	[93]	CEA, Cost description	Mass dog vaccination campaign in 2012 and 2013	Number of vaccinated animals (dogs, cats, primates), rabies cases, vaccination cost	Operational performance of rabies vaccination for dogs
Wallace et al.	2017	[94]	CEA, Economic Framework	Interviews, vaccination campaigns	Years for rabies elimination, country development status, dog vaccination availability, and costs, existing animal health workers	Resource needs, feasibility for global elimination of dog-mediated human rabies
Gibson et al.	2022	[65]	CEA, Logistic regression analysis, Burden of disease	Dog vaccination, rabies education, RabiesEcon (model for CE)	Dependent variable: Rabies case Independent variables: 12-month vaccination coverage, dog population density Cost per death averted, DALY	Feasibility for elimination of human rabies, CE of vaccination for human rabies
Acquisto et al.	2022	[97]	CMA, Wilcoxon Rank Sum test, Chi-Squared analysis, Fisher's Exact Test	Retrospective observational study, immune globulin dose	Cost savings for rounded dose of IGD (amount of being wasted), rate of re-presentation of patients, rabies infection	CE and waste reduction through immune globulin against rabies in humans
Babo Martins et al.	2022	[70]	COI, Burden of disease	Survey	DALYs, livelihood losses, out-of-pocket healthcare expenditures, losses due to mortality/productivity due to days off work, treatment costs in domestic animals	Quantify the health and socioeconomic burden of snakebites in humans and domestic animals
Freddy Patrick et al.	2024	[98]	COI, Burden of disease	Retrospective analysis in three cities (vaccination, hospital records, surveys) for estimation of costs over 10 years	Incidence, costs for preventive measures, vaccination, corrective procedures (Post Exposure Treatment), DALYs, societal cost of disease	Socio-economic burden of rabies in humans and animals, focuses on direct costs
Dupont et al.	2022	[52]	COI, Burden of disease, Monte-Carlo simulation, Markov-Chain Model	Use of different datasets	DALYs, cost of illness, direct healthcare costs, patient costs, productivity losses, incidence	CE of surveillance of Neurocysticercosis in humans
Cassini et al.	2022	[59]	COI, Integrated epidemiologic and economic model	Expert opinions, use of different datasets,	Hospitalization costs, productivity losses, Consumer Price Index	Costs of cystic echinococcosis in humans and cattle
Ferguson et al.	2020	[47]	Cost estimation approach	Mass dog vaccination campaign, questionnaires	Cost per vaccinated dog, vaccination coverage rate, number of dogs vaccinated, operational feasibility, KAP: rabies, dog vaccination, community engagement	Operational performance, evaluate feasibility of rabies vaccination for dogs
Rysava et al.	2019	[96]	Cost estimation approach: analysis framework	Bite patient risk assessment against rabies	Costs of rabies post-exposure prophylaxis for 3 scenarios	CE surveillance against rabies in humans
Bodenham et al.	2021	[39]	Cost estimation approach: Direct cost analysis	Questionnaire, Outbreak costing tool	7 cost categories: labor, office, travel and transport, communication, laboratory support, medical countermeasures, and consultancies	Costs for Anthrax outbreak in humans and animals
Naïssengar et al.	2021	[95]	Cost estimation approach: Direct cost analysis	Decentralized animal rabies surveillance: Staff training, sample transport, community engagement	Proportion of positive samples, costs for diagnostic units, barriers for implementation	Evaluate feasibility, challenges, and impact of rabies in humans
Lin et al.	2019	[69]	Dynamic panel data model (with Regression analysis), Principal component analysis	Use of different datasets	Time series plot of human incidence in 3 clusters, correlating socio-economic factors	Effectiveness of risk factors of human brucellosis
Mummah et al.	2020	[140]	Economic framework	Mathematical model	Incidence, spillover risk, relative costs for control measures	Relative effectiveness in reducing human-to-human transmission of different zoonoses
Pendell et al.	2016	[78]	Economic-epidemiological model	Survey for WTP, modeled outbreak response measures	Quantified economic losses under different outbreak scenarios (estimation of impacts on agricultural producers and consumers, government costs of response, costs, and disruptions to non-agricultural activities, human morbidity, and mortality)	Costs for Rift Valley Fever in livestock and humans
Berlinguer et al.	2021	[73]	Ecosystem services evaluation	Feeding station for vultures with livestock cadavers	Incineration costs, CO2 costs, total animal biomass provided/ consumed in kg	CE of vulture feeding stations for controlling Cystic Echinococcosis
Canali et al.	2020	[61]	Epidemiologic-economic models	Research of experts, Policy guidance for surveillance	Flow charts with cost categories and epidemiologic outcomes	Costs of cystic echinococcosis and leishmaniasis in animals and humans
Da Gonçalves et al.	2024	[88]	Generalized linear mixed models	Collecting insects, census	Income, infection rate (other sociodemographic variables)	Socio-environmental factors influencing triatomines in humans
Bonaparte et al.	2023	[138]	Global Framework for the Elimination of dog mediated rabies (STOP-R- Index)	Use of different datasets	Index (0−100) for human rabies deaths (indicators: Literacy rate, infant mortality rate, electricity access, political stability, presence/severity of natural hazards)	Effectiveness of rabies elimination in humans
Agampodi et al.	2023	[76]	Indirect COI, Burden of disease	Use of different datasets	Loss of productivity DALYs, GDP, health expenditure	Economic burden of Leptospirosis in humans
Ochoa et al.	2021	[71]	Integrated Nested Laplace Approximation Models	Survey, observations, geospatial analysis	Snakebite risk model, maps of snakebite risks, (including e.g., Poverty probability Index, Normalized Difference Vegetation Index, distance to water)	Effectiveness of snakebite prevention in humans and animals
Almanfaluthi et al.	2022	[77]	Linear regression, Principal Component analysis, Bayesian disease mapping	Survey, parasitological diagnosis	Regional comparison, risk factors to disease (e.g., GDP, income)	Risk factors of Opisthorchiasis and Leptospirosis in humans
Lokamar et al.	2020	[46]	Logistic regression	Surveys, interviews	Dependent variable: Stillbirth Independent variables: e.g., sale value, cost of treatment	Socio-economic impact of Brucellosis on livestock production for Pastoralists
Mtui-Malamsha et al.	2019	[38]	Logistic regression	Vaccination of pets, questionnaire, interview, serum samples of pets	Dependent variable: seroconversion status Independent variables: e.g., the proportion of dogs and cats in the household; qual. finding, e.g., costs and distance as barriers against vaccination	Effectiveness of vaccination against rabies in humans and animals
Hobbs et al.	2018	[80]	Logistic regression, *t*-test	Education workshop, questionnaires	Dependent variable: Questionnaire scores pre and post Independent variables: e.g., gender, age, study neighborhood	Effectiveness of an educational program about Taenia solium for primary school students
Cociancic et al.	2020	[75]	Logistic regression, Chi-square, Fisher's-exact	Questionnaire, collection of stool samples, parasitological analysis	Dependent variable: Infection status Independent variables: e.g., governmental monetary support, parents' employment	Risk factors of intestinal parasitosis in children and youth
Benitez et al.	2017	[83]	Logistic regression, cluster analysis, kernel-smoothed intensity analysis	Questionnaires, blood samplings	Dependent variable: Seroprevalence Independent variables: e.g., monthly income, meat consumption	Risk factors and Toxoplasma gondii antibodies in owners and dogs
Savadogo et al.	2022	[87]	Mixed-methods: Causal loop analysis, qual. Analysis	Individual and focus group in-depth Interviews	Interconnected factors influencing rabies, vaccination costs	Effectiveness of rabies dog vaccination
Häsler et al.	2014	[86]	Mixed-methods: CEA, CBA, Burden of disease in epidemiological economic modeling, qual. Analysis	Mass vaccination, sterilization of dogs, community education, surveys	Program costs, DALYs, treatment, labor, medication reduced culling, social acceptance, animal welfare	Evaluation of vaccination against rabies in humans and dogs
Lankester et al.	2019	[37]	Mixed-methods: CEA, qual. Analysis	Vaccination for dogs, deworming school-aged children, interviews, group discussion	Participation rate, cost savings compared to other interventions	Feasibility, CE of vaccination for soil-transmitted helminths and canine-mediated human rabies
Mpolya et al.	2017	[42]	Mixed-methods: CEA, qual. Analysis	Mass dog vaccination campaign, surveys, interviews	Cost-per-dog vaccinated, per life saved, for administration of post-exposure prophylaxis, vaccination coverage, bite incidence, human rabies deaths, policy impact	Feasibility, CE of rabies vaccination for dogs
Grace et al.	2016	[44]	Mixed-methods: Ecosystem-poverty-health framework, qual. Analysis	Survey	Direct and indirect health costs (e.g., spending on preventive care for humans and animals)	CE vaccination for pastoralists against Rift Valley fever
Voupawoe et al.	2020	[84]	Mixed-methods: Global Dog Rabies Elimination Pathway (GDREP) Tool, qual. Analysis	Mass dog vaccination, four rabies diagnostic tests, workshop	Diagnostic confirmation of rabies cases in animals, costs per mass dog vaccination over 13 years, time till the elimination of human rabies death	CE rabies diagnostic and vaccination in dogs
Sun et al.	2024	[91]	Mixed-methods: Global One Health Index (GOHI)	Questionnaires, interviews	Scale (0–100): 5 key indicators, 16 sub-indicators (e.g., GNI, HDI)	Effectiveness of surveillance for zoonoses in humans and animals
N'Guessan et al.	2022	[99]	Mixed-methods: Linear regression, qual. Analysis	Questionnaires, observations, interviews, group discussions	Dependent variable: Treatment completion Independent variables: e.g., income, age, education level; Determinants of PEP Drop-Out	Risk factors for human drop-out rate of rabies post-exposure prophylaxis
Barnes et al.	2018	[48]	Mixed-methods: Logistic regression, qual. Analysis	Survey	Dependent variable: Animal ownership Independent variables: e.g., wealth tercile, number of people in the household	Risk factors for zoonoses in humans
Marchino et al.	2021	[62]	Mixed-methods: qual. Analysis, process evaluation	Group discussion, questionnaires	Satisfaction score for surveillance (1 = totally unsatisfied, 10 = fully satisfied), open questions (e.g., strength as CE), timelines	Effectiveness of public health policy for West Nile virus in humans
Buttigieg et al.	2018	[74]	Mixed-methods: qual. and quant. Analysis with NEOH tool	Vaccination, education, surveillance	OH Index (0–1)	Effectiveness of vaccination Brucellosis in humans and animals
Garcia-Vozmediano et al.	2022	[58]	Mixed-methods: qual. and quant. Analysis with NEOH tool	Collection of reports, discussion	OH Index (0–1)	Effectiveness of surveillance of tick-borne diseases for animals and humans
Hanin et al.	2018	[36]	Mixed-methods: qual. and quant. Analysis with NEOH tool	Interviews, surveys	OH Index (0–1)	Effectiveness of surveillance of Infectious Diseases for humans and animals
Laing et al.	2018	[82]	Mixed-methods: qual. and quant. Analysis with NEOH tool	(Retrospective) application of acaricides	OH Index (0–1): scores for OH thinking, planning, and working, planning	Effectiveness of pesticides against tick-borne diseases in cattle
Wang et al.	2019	[67]	Mixed-methods: Qualit. analysis, ranking with One Health Zoonotic Disease Prioritization (OHZDP)	Workshop	30 diseases ranked (incl. disease hazard/severity case-fatality in humans, epidemic scale & intensity in animals & humans, economic impact, prevention and control, social impact)	Effectiveness of surveillance of zoonoses in humans and animals
Alcoba et al.	2021	[72]	Mixed-methods: quant. and qual. Analysis	Survey	District-level incidence rates, mortality, expenditure, and productivity loss, Poverty Probability Index	Costs of snakebites in humans (primary data)
Goryoka et al.	2021	[92]	Mixed-methods: ranking process (OHZDP), qual. Analysis	Workshop, discussion	Scoring list of 7 zoonoses (socio-economic impact)	Effectiveness of surveillance of zoonotic diseases
Coffin et al.	2014	[50]	Mixed-methods: Ranking, weighting Chi-square test, qual. Analysis	Questionnaire, interviews, discussions, observations	Ranking of tick-borne diseases, risk factors (e.g., proximity to outbreak/National Park)	Prioritize tick-borne diseases in humans and animals
Sakshi et al.	2023	[64]	Mixed-methods: Scoring system based on One Health Zoonotic Disease Prioritization (OHZDP)	Workshop, focus group discussion, interviews	7 criteria with weightage score (e.g., socio-economic burden, prevalence, bioterrorism potential) ranking of 15 zoonoses	Prioritize zoonotic diseases in humans and animals
Adeyemo et al.	2022	[79]	Mixed-methods: Stochastic partial budget models, qual. Analysis	Survey, group discussions	Median disease costs per year per head of cattle, sheep, and goats	CE test and treatment for livestock with schistosomiasis
Jato-Espino et al.	2023	[141]	Multi-criteria decision analysis	Open-source urban indicator computational framework, weighting with Entropy method	Composite Index: range of values for risk disease spread (e.g., average income)	Model zoonotic disease spread
Acosta et al.	2019	[53]	Qual. analysis	Observations, focus group discussion, interviews	Supply chain challenges, vaccination costs, lead times of level of the supply chain	Effectiveness of supply chain vaccination against peste de petits in ruminants
Mbaipago et al.	2022	[89]	Qual. analysis	Workshops, interviews	Risk factors (low purchasing power, transportation costs, distance to vaccination)	Effectiveness of dog vaccination against rabies
Osmani et al.	2021	[90]	Qual. Analysis	Questionnaires	KAP (e.g. no regular vaccination program for cattle)	KAP of farmers, animal traders, veterinary professionals regarding Foot and Mouth Disease
Sichewo et al.	2020	[85]	Qual. analysis	Focus group discussions	KAP (e.g., economic pressure leads to risky animal practices)	KAP of farmers and household members handling tuberculosis in livestock and humans
Stefania et al.	2024	[63]	Qual. analysis	Interview, surveys	KAP, risk factors against biosecurity (e.g., medication costs)	KAP of farmers handling avian influenza in turkeys
Thys et al.	2016	[81]	Qual. analysis	Survey, focus group discussions	KAP: cultural, economic, and gender (risk)factors (such as costs for vaccination)	Effectiveness of management of pig farming in reducing Taenia solium for people
Munyua et al.	2016	[45]	Ranking and scoring tool (OHZDP), Burden of disease	Survey, discussion	A ranking list of 36 zoonoses, decision tree was used to score each zoonotic disease (information about DALYs, mortality, and others was given before)	Effectiveness of surveillance of zoonotic diseases
Decker et al.	2021	[41]	Stated preference choice analysis	Survey	Willingness to pay, Probability of vaccine uptake, predictors of incidence	CE of vaccination cattle against malignant catarrhal fever
Meisner et al.	2019	[49]	Stochastic compartmental and deterministic model	Mathematical model	Incidence, prevalence, deaths	Effectiveness of trypanocides treatment against sleeping sickness in humans, fly vectors, and cattle
Meisner et al.	2023	[51]	Stochastic compartmental model	Model for insecticide/drug treatment for animals	Probability of and time to elimination of transmission	Effectiveness of insecticide and medication for human Sleeping sickness

70 % of the studies were conducted at the regional level (*n* = 43), mostly in African countries (51 %; *n* = 22). A total of 49 % of the studies undertaken at the regional level had a rural area focus (*n* = 21), 23 % were carried out in urban and rural areas (*n* = 10), and 14 % in urban and/or peri-urban areas (*n* = 6).

The majority of papers, around 34 %, used mixed-methods with qualitative and quantitative analysis (n = 21). Qualitative data were collected through group discussions, interviews, surveys, observations, and workshops, and were used for the understanding of influencing factors leading to zoonosis or for the implementation of interventions [[86], [87], [88]]. Qualitative research helped to identify barriers to vaccination, such as distance and cultural beliefs [89]. Knowledge, Attitudes, and Practices (KAP) were analyzed in 8 % of the studies. KAP focused on the interaction between farmers or pet owners and their interaction with turkeys, sheep, pigs, cattle, and dogs to analyze the surveillance for zoonoses (*n* = 5) [90]. A One Health Index based on a mixed method approach from the Network for Evaluation of One Health tools (NEOH) (*n* = 4), was used to evaluate on a scale from 0 to 1, a score for One Health thinking, planning, working, sharing, learning, and systemic organization [36,58,74,82]. The goal of those studies was to analyze the Effectiveness of surveillance through vaccination or pesticides against diseases in humans and animals. Data for the One Health Index was gathered through interviews, surveys, discussions, and existing reports. In one study, the Global One Health Index (GOHI) compared zoonoses prevention and control for different countries, taking into account “case studies, capacity building, target population, route of transmission, and source of infection” [91]. The Human Development Index was a significant parameter for the GOHI. Four studies [45,64,67,92] employed the One Health Zoonotic Disease Prioritization (OHZDP) tool, which facilitates the assessment of diverse diseases. This assessment encompasses various dimensions, including disease hazard/severity, case-fatality in humans, epidemic scale and intensity in animals and humans, economic impact, prevention and control, social impact, and bioterrorism potential. Additionally, the OHZDP was utilized for scoring diseases of a similar nature (e.g., tick-borne diseases) [67].

With approximately 15 %, CEA was the most frequently used quantitative economic analysis for zoonoses (*n* = 9) for details, see Table 2. These studies aimed to analyze the CE and feasibility of vaccination and surveillance programs. Outcomes were the cost of vaccination/administration, cost savings compared to other interventions, number of vaccinations, incidence, deaths, DALYs, and YLG. [37,40,42,43,60,65,86,93,94].

In the minority (*n* = 4), cost estimation approaches were performed [39,47,95,96]. These studies also analyzed costs, as did the CEA, but with a partial approach. Those studies analyzed the costs of implementing surveillance and vaccination programs in a more limited scope than a full CEA, with the main objectives being to support program implementation and to assess cost aspects [47,96,97]. In another study, the costs of laboratory, office, travel and transport, communication, laboratory support, medical countermeasures, and advice were recorded to illustrate the costs of an anthrax outbreak [39]. In a decentralized rabies surveillance program, the collection of diagnostic units and the proportion of samples testing positive were used to study the impact of rabies [95].

In only one study, a CMA was performed to evaluated the cost savings and reduction in waste of rabies immune globulin by comparing rounded versus non-rounded dosing strategies, and monitored patient re-presentation rates [97]. CBA was conducted in one study each. In the study with CBA, a CEA was incorporated into an epidemiological economic model [86]. The aim was to evaluate rabies vaccination, including program costs, DALYS, social acceptance, and animal welfare.

Socio-economic costs were captured by the COI, including for the evaluation of surveillance systems and the assessment of the socio-economic burden of zoonoses (*n* = 4). For example, direct health care costs included out-of-pocket expenditures, treatment costs, and preventive measures costs. [52,59,70,98].

For burden of disease, DALYs were used in 15 % of the zoonoses studies (*n* = 9), mainly used to quantify the impact of zoonoses, such as rabies, snakebite, Neurocysticercosis, and Leptospirosis, on humans and animals [40,43,45,52,65,70,76,86,98]. Loss of livelihood, loss of productivity, and other societal costs of disease have been used to analyze the impact of different zoonoses on humans and animals [52,70,98]. Indirect costs have been analyzed, for example, by measuring lost productivity or by assessing changes in GDP as a macroeconomic indicator [76].

As a statistical approach, logistic regression was the most frequently used method, with 10 % (*n* = 6) for measuring impacts on zoonoses [38,46,48,75,80,83]. The study aims were to analyze risk factors for zoonoses [48], socio-economic impacts [46], the CE of vaccination [65] and the effectiveness of educational programs [80]. Binary dependent variables served statuses such as seroprevalence, infection, increased post score in the questionnaire, monthly income, or meat consumption. In addition to social variables (gender, age, sex, parents' employment), economic variables were considered, including the cost for treatment, two-month vaccination coverage, the sale value of an animal, dog population density, cost per death averted, DALYs, wealth tercile, number of people and animals in the household, and governmental monetary support. The same variables served as dependent or independent variables in linear regression (5 %), apart from one additional parameter: treatment completion as a dependent variable (*n* = 3).[68,77,99].

Through ranking and scoring, zoonoses were weighted and prioritized for surveillance in 8 % of the studies [45,50,64,67,92]. Causal loop analysis was used in one study to explore and map the complex dynamics underlying low dog rabies vaccination coverage, specifically aiming to identify barriers and facilitators to effective zoonosis control in a low-income setting [87]. One study used a Markov model (DisMod II) to estimate age-stratified epidemiological parameters for neurocysticercosis-associated epilepsy, and a Monte Carlo simulation to quantify the uncertainty in their health and economic impact estimates by generating confidence intervals through 100,000 simulations [52].

In approximately less than 5 % of the studies, Stochastic models [49,51,79], chi-square test [50,75,97], Principal component analysis [69,77], Bayesian disease mapping [77] and Integrated Nested Laplace approximation models [71] were used for statistical analyses and modeling approaches.

### 3.3 Economic approaches and geographical context for articles focusing on AMR

After zoonoses, AMR was the second most common topic in the studies (14 %, *n* = 15), see Fig. 4. In the AMR studies found, most of the studies (40 %, *n* = 6) were conducted at a regional level [66,[100], [101], [102], [103], [104]]. However, no specific country or continent focus could be identified. Of all AMR papers, 14 % addressed the effectiveness and evaluation of various surveillance tools (*n* = 7) [55,[104], [105], [106], [107], [108], [109]], risk factors for AMR (*n* = 2) [66,101], and other topics related to laboratory performance [110], antibiotic dosages for animals and plants [54,100], differences in antibiotic purchases and prescribing [102,103], see Table 3. One study used a causal loop diagram to illustrate the complex interaction for effective AMR surveillance in the food chain [108]. An agricultural human health micro-economic model was constructed based on a Markov model and CUA and CBA [111]. This model was used to evaluate the cost-effectiveness of AMR in food production, focusing on several parameters, including willingness to pay, QALYs, and labor productivity. Benchmarking was employed in the form of an evaluation tool (OH-EpiCap), where participants scored impact, organization, and operation [109]. A SWOT analysis of different evaluation tools revealed that each tool can be used for other goals (such as AMR surveillance or animal health), but all require expertise in veterinary and epidemiological understanding [105]. In a GOHI-AMR, an extended version of GOHI, various indicators, such as GDP and life expectancy, were used to rank countries on their AMR handling [107].

**Table 3:** Economic One Health approaches for antimicrobial resistance (AMR).

Author	Year	Source	Economic approach	Intervention	Outcome	Study aim
Lazzarino et al.	2021	[54]	Benchmarking	Sivar software	Defined Daily Dose for antibiotic (mg/kg/day)	Antibiotic dosage for beef
Zhou et al.	2022	[107]	Global One Health Index (GOHI-AMR)	Discussions	Score (0–100): 5 key indicators, 17 indicators (e.g., GDP, Life expectancy)	Effectiveness of AMR surveillance
Rousham et al.	2023	[102]	Logistic regression	Observations and a short interview	Dependent variable: Prescription use for antibiotics Independent variables: e.g., animal owners, type of drug shop	Gender and urban-rural dimensions for antibiotic purchase
Emes et al.	2023	[111]	Markov chain transition model, CEA, CBA	Agriculture Human Health Micro-Economic model (AHHME)	Timeframe (years), discount rate, willingness to pay per QALY, labor productivity and annual growth rate, the ratio of paid work to total (paid/unpaid) work, cost of providing a hospital bed for one day, other non-economic parameters	CE AMR interventions in food production
Norström et al.	2023	[109]	Mixed-methods: Benchmarking, qual. Analysis	OH-EpiCap: Evaluation tool, interviews	Score (1 = no compliance to 4 = full compliance) for impact, organization, operation	Effectiveness of AMR surveillance tool
Mathew et al.	2024	[66]	Mixed-methods: qual. Analysis, Ranking, Scoring	Local framework testing, expert feedback	15-point indicator system	Risk factors for AMR
Abegaz et al.	2023	[110]	Mixed-methods: quant., qual. Analysis for indirect cost influences	Questionnaires, interviews, External Quality Assessment	Challenges and opportunities of virtual laboratory assessment	Effectiveness of laboratory performance
Manderson et al.	2020	[103]	Qual. analysis	Interviews	Details about antibiotic prescriptions (e.g., economic status)	Socio-economic context of antibiotic prescribing
Asaduzzaman et al.	2024	[104]	Qual. analysis	Interviews, District Health Information System (DHIS2)	Stakeholder viewpoints	Effectiveness of AMR surveillance platform
Lambraki et al.	2023	[108]	Qual. analysis, Causal Loop diagram	Workshops, interviews	98 factors with 362 connections	Effectiveness of AMR surveillance in the food system
Haworth-Brockman et al.	2021	[106]	Qual. analysis, One Health Evaluation of Antimicrobial Use and Resistance Surveillance (OH-AMURS)	Interviews	Tool for strength and weakness of AMR surveillance, evaluation matrix with 36 components	Effectiveness of AMR surveillance
Moura et al.	2023	[55]	Qual. analysis: SWOT	OH-EpiCap	Strengths, Weaknesses, Opportunities, Threats	Evaluation of AMR surveillance tool
Nielsen et al.	2020	[105]	Qual. analysis: SWOT, Scoring	Progressive Management Pathway tool on AMR (AMR-PMP), Network for Evaluation of One Health (NEOH), Integrated evaluation of animal health surveillance systems (SURVTOOLS)	Strengths, Weaknesses, Opportunities, Threats, Score for content, evaluation, stakeholder engagement, resource demand, integration, applicability	Effectiveness of three AMR surveillance tools
Paumier et al.	2022	[101]	Quasi-Poisson regression models	Isolation of *E. coli* from community urine samples	Risk factors for infection (e.g., social deprivation Index based on non-employed for population 16–64 years in percent, vehicles per household)	Risk factors for *E. coli* in urinary tract infections
Chanvatik et al.	2019	[100]	Survey assessment, productivity loss	Questionnaire, field visits	Dose of antibiotic 1 mg/L water, qual. Information about e.g., production cost, production, and market volume	Risk factors for greening disease and antibiotic use in Mandarin production

### 3.4 Economic approaches and geographical context for articles focusing on food safety and security

The third largest category, with 7 %, concerned food safety and security (*n* = 8), with two studies specifically targeting the bushmeat markets in Laos [112,113]. Half of the studies (*n* = 4) were conducted at the regional level [35,112,114,115], with three of them focusing on rural areas [35,114,115]. Articles in this field included risk factors and surveillance for AMR in food [35,116], for infectious diseases and zoonoses in animals [35,[112], [113], [114]], and animal and plant health through sustainable and nature-based approaches [115,117], see Table 4. The studies employed various approaches to evaluate food safety and security, including causal loop diagrams, fuzzy analytical hierarchy processes, and multinomial logit models. For sustainable food safety and security, GOHI-FS indicators on three levels of investment and financial support scores were used, including, e.g., GDP per capita, health expenditure, HDI, and life expectancy [118]. The results of these studies highlight the importance of a comprehensive analysis of risk factors and surveillance measures for AMR, infectious diseases, and zoonoses in food safety and security, to protect public health and animal health.

**Table 4:** Economic One Health approaches for food safety and security.

Author	Year	Source	Economic approach	Intervention	Outcome	Study aim
Young et al.	2014	[114]	Generalized linear mixed models, Logistic regression, qual. Analysis	5-year project: Questionnaire, Training in vaccination, forage development, and husbandry	KAP of smallholder cattle farmers, Dependent variable: (no) Increase annual household income Independent variables: household income, time saving, livelihoods	Enhance food security and improve cattle health through husbandry practices
Varyvoda et al.	2024	[115]	Mixed method single case study: quant. and qual. Analysis	Questionnaires, interviews	40 nature-based solutions for food systems, ranking of resources for implementation	Nature-based approaches for small-scale farmers, ranchers, and food entrepreneurs
Gu et al.	2023	[118]	Mixed-methods: qual. Analysis, Fuzzy Analytical Hierarchy Process, Linear regression	Global One Health Index-Food Security (GOHI-FS), interviews	Indicators on 3 levels (e.g., investment and financial support score), Regression for the relation of GOHI-FS and e.g., GDP per capita, health expenditure, HDI, life expectancy	Tool for sustainability in food security
Nthambi et al.	2023	[35]	Mixed-methods: qual. Analysis, Stated choice experiment, Multinomial logit model	Survey, Choice behavior of farmers e.g., disease scenarios, effects of previous disease experience, education level, and income)	Qual. results, e.g., treatment decisions vary across livestock species, policy recommendations, awareness-raising programs	Preferences for livestock disease treatment (AMR)
Philavong et al.	2020	[112]	Multinominal logistic regression	Survey, observations	Dependent variable: low/high health risk Independent variables: e.g., vendor types (Vegetable, livestock, and wildlife meat), trade as main occupation	Risk factors for infectious diseases among different market vendors
Lambraki et al.	2022	[116]	Qual. analysis, Causal loop diagram	Workshop with stakeholders	91 factors, 331 connections influencing AMR in food systems (e.g., places for interventions)	Effectiveness of surveillance of AMR in food systems
Pruvot et al.	2019	[113]	Quantitative risk analysis	Consumer and vendor surveys, observations	24 factors (e.g., proportion of sold bushmeat, yearly frequency of consumption)	Risk factors for zoonoses transmission at bush meat markets
Savary et al.	2021	[117]	Various stat. Analysis: e.g., Hierarchical cluster, Principal component, Multiple regression	Retrospective surveys, use of different datasets, e.g., the Office of Agricultural Economics	Risk factors for rice yield reduction (e.g., injury profiles of plants through diseases, pests, weeds)	Effectiveness of rice health (plant diseases)

### 3.5 Economic approaches and geographical context for articles focusing on animal welfare

A focus on animal welfare was found in 6 % of all studies (*n* = 7), see Table 5. Four of those studies were conducted at the regional level [[119], [120], [121], [122]] of which three were conducted in urban and/or peri-urban areas [119,120,122]. The articles showed considerable diversity in their objectives, including integrating animal health into the UN Sustainable Development Goals [123], improving the health of companion and working animals [[119], [120], [121]], protecting avian biodiversity for general population health [56,124], and promoting access to veterinary services [122]. This broad approach was also reflected in the economic methods used, such as canonical correspondence analysis, linear and multivariate regression, generalized linear models, causal analysis, spatial analysis, and trajectory analysis. Qualitative analysis was also used to investigate the complex relationships between animal health and economic factors [119].

**Table 5:** Economic One Health approaches for animal welfare.

Author	Year	Source	Economic approach	Intervention	Outcome	Study aim
De Moura et al.	2022	[120]	Canonical correspondence analysis, Generalized linear model	Survey	Hoarding and animal protection profile, socio-economic risk factors (e.g., income per capita, HDI)	Effectiveness of animal protection
Bjørnvad et al.	2019	[121]	Linear regression, logistic regression	Questionnaire	Risk factors for obesity in companion dogs and owners (e.g., GDP, monthly family income)	Prevention of obesity in humans and dogs
Keeling et al.	2022	[123]	Mixed method: Scoring and qual. Analysis	Workshop, group discussion	Score (−3; +3) for the strength of improvement animal welfare and achieving SDG (e.g., economic growth)	Integrating animal welfare into the UN Sustainable Development Goals (SDGs)
De Klerk et al.	2020	[119]	Mixed method: Spatial analysis and qual. Analysis	Questionnaire	Income sources, household support, socio-economic conditions (e.g., earnings per day)	Socio-economic impacts of working horses
Chen et al.	2023	[124]	Multivariate regression	Use of different datasets, e. g., citizen science bird data	Life expectancy at birth, age-specific mortality risk (associated with a higher number of bird species)	Considering animal biodiversity for public health
Reese and Li	2023	[122]	Multivariate regression	Use of different datasets, e.g., Geographical information system, census data	Dependent variable: Number of pet stores and veterinarians per capita Independent variables: poverty levels, residential economic health Index, Need for Animal Support Services Index)	Effectiveness of access to animal welfare resources considering socio-economic conditions
Nadal et al.	2024	[56]	Trajectory analysis, Linear models	Use of different datasets, e.g., historical migration patterns, urbanization metrics	Bird recovery rates (influenced, e.g., by GDP, cereal, and legume production)	Ecological impacts (on birds) of urbanization

### 3.6 Economic approaches and geographical context for articles focusing on governance

The topic of governance was covered in 6 % of the studies (*n* = 7), see Table 6. The studies in the field of governance were performed on a global (*n* = 2) [125,126], multi-country (n = 2) [127,128], and on the national level (*n* = 3) [[129], [130], [131]], without an area specification. Five of them employed an economic approach based on ranking [[125], [126], [127], [128], [129],^131^]. The ranking's objectives included enhanced surveillance of zoonoses through the prioritization of the most significant, for example, classified according to cost and impact [129]. The One Health Governance Index and the Global One Health Integrated Drivers Index, which take into account factors such as World Bank income and policy effectiveness, were used as additional ranking methods [126]. In addition, the SWOT analysis served to identify strengths and weaknesses in two articles, once for the evaluation of a national AMR report [130] and in a second study on the prioritization of One Health initiatives [128].

**Table 6:** Economic One Health approaches for governance.

Author	Year	Source	Economic approach	Intervention	Outcome	Study aim
Degeling et al.	2017	[131]	Mixed method: Ranking and qual. Analysis	Online Delphi survey	19 surveillance actions ranked by experts (e.g., economic impacts), qual. Opinions on OH definition, collaboration	Improve experts' surveillance against zoonoses
Cediel Becerra et al.	2021	[127]	Mixed method: Ranking and qual. Analysis	Online survey	Rating implementation scores (1 = poor, 5 = excellent) for perception, knowledge, awareness of OH, qual. Results about collaboration	Improve intersectoral collaboration
Cargnel et al.	2024	[130]	Qual. analysis, Stakeholder quadrant analysis, SWOT	Belgium's annual national OH report (BELMAP)	Key organizations, strengths, and weaknesses of the report	Effectiveness of AMR report
Guo et al.	2023	[125]	Ranking, Correlation	Global One Health Integrated Drivers Index (GOH-IDI)	3 first-level, 15 second-level, 61 third-level indicators (e.g., World Bank income groups)	Global governance capacity
Li et al.	2023	[126]	Ranking, fuzzy analytical hierarchy process	One Health Governance Index (OHGI)	Score (0 = lowest to 100 = highest) based on weights for 8 indicators, 19 sub-indicators (e.g., effectiveness and efficiency)	Global governance capacity
Ndoungué et al.	2023	[129]	Ranking, qual. Analysis	National Bridging Workshop	55 ranked actions (e.g., by cost and impact)	Effectiveness of the coordination tool against zoonoses
Fasina et al.	2021	[128]	Stakeholder quadrant analysis, SWOT, Ranking	Questionnaire and online survey	Overview of OH networks, challenges, ranking OH initiatives (1 = low to 5 = high)	Map of OH landscape in Africa

### 3.7 Economic approaches and geographical context for articles focusing on veterinarians

The topic of veterinarians was the focus of 4 % of the articles (*n* = 4), see Table 7. Three of the studies were conducted at the national level and underscored the necessity for enhanced training of veterinarians and their understanding of zoonoses [[132], [133], [134]]. Of the four studies, two (one conducted in Vietnam and the other in Ethiopia) employed a statistical analysis to evaluate the training of veterinarians [132,134]. In the study in Ethiopia, veterinarians assessed the economic relevance of zoonoses and the obstacles that arise when implementing training programs [134]. In addition, a mixed-methods approach was used to analyze veterinarians' knowledge of biosecurity, taking into account experience with One Health, years in the profession, and education as factors [132]. Another study at a regional level in Australia offered field trips where participants could learn in the field how to recognize and control specific zoonotic species and then evaluate their experiences [135]. The results of these studies show that training veterinarians and improving their knowledge of zoonoses are crucial to protecting public and animal health. In a further study, conducted in the U.S.A., veterinarians in zoos were asked to what extent conversation medicine and One Health were already being used in their workplaces, the zoos, and aquariums, based on the expenditure on corresponding measures and the size of the facilities, among other things [133].

**Table 7:** Economic One Health approaches for veterinarians, environment and other topics.

Economic One Health approaches for veterinarians						
Author	Year	Source	Economic approach	Intervention	Outcome	Study aim
Auplish et al.	2024	[132]	Mixed method: qual. Analysis, logistic regression models, descriptive analyses	Online survey, semi-structured interviews	KAP biosecurity of veterinarians, barriers such as resources and funds of small farms, Dependent variable: experience in One Health practice Independent variables: education, years worked	KAP biosecurity of veterinarians
Mor et al.	2018	[135]	Rating, chi-square tests	Online survey, field trips with stations for different zoonoses (in the curriculum of veterinary students)	Rating of students for 5 stations (Likert scale from very poor to very good)	Implementing OH in veterinary medical students' curriculum
Sulzner et al.	2021	[133]	Semi-quantitative survey, Fisher's exact test	Survey	Engagement of zoo veterinarians in conservation medicine/OH (such as size, spending)	Scope of Conservation Medicine/OH in zoos/aquariums
Alafiatayo et al.	2022	[134]	Scoring, descriptive statistical analysis	Survey	Valuation of veterinarians on e.g., training topics, economic relevance of diseases, barriers of service delivery, such as lack of equipment, unavailability of governmental funds	Effectiveness training of veterinarians

Economic One Health approaches for the environment						
Author	Year	Source	Economic approach	Intervention	Outcome	Study aim
Fania et al.	2024	[57]	AI model (based on Shapley additive explanation, Linear Model, Random Forest Model, Extreme Gradient Boosting)	Use of different datasets	Estimation of daily ground-level concentrations of air pollutants, factors in model such as temperature, population density, wind speed	Effectiveness surveillance of air pollution
Zhou et al.	2022	[68]	Fixed effects regression model (multiple regression)	Use of different datasets	Dependent variable: incidence of Malaria Independent variables: e.g., diesel fuel, pesticides, toilet investment, income, temperature	Analysis of zoonoses and environmental pollution
Akuoko et al.	2023	[136]	Mixed-methods: qual. Analysis, Socio-economic framework	Surveys, focus-group interviews	Factors influencing human well-being, such as economic security (employment, income), environmental quality	Analysis of well-being and consequences of plastic waste

*Economic One Health approaches for other topics*						
Author	Year	Source	Economic approach	Intervention	Outcome	Study aim
Johnson et al.	2019	[142]	Discrete choice experiment, mixed logit model	Web-based survey, Online panel with 18 scenarios, such as economic development (unemployment)	Public preferences: willingness to pay, differences in prioritization of interventions	Analysis of public preferences in emerging infectious diseases
McIntyre et al.	2019	[143]	EuroQol-5D-3L, CUA, CE, burden of disease	Real-time detection, diagnosis, and control system, questionnaire	Framework for new surveillance system (e.g., setup costs, QALYs)	Effectiveness of surveillance of diarrheal disease
Ionescu et al.	2022	[144]	Mixed method: review, SWOT, econometric model, linear regression, and other statistical analyses	Literature review, survey	A model with relations and factors for management decisions in tourism, Dependent variable: GDP in tourism Independent variables: e.g., decrease in purchasing power, unemployment rising	Tourism decision support model after a pandemic

### 3.8 Economic approaches and geographical context for articles focusing on the environment

Studies with a focus on the topic environment were the fewest found, with 3 % (*n* = 3), ***see*** Table 7. One study was conducted at a regional level in Italy in an urban and rural area [57]. The study investigated the effects of air pollution on human health and used an AI model to calculate the daily concentrations of air pollutants, factors such as temperature, population density, and wind speed, using satellite and climate data. A study in a rural area in China investigated the environmental impact of zoonoses using the example of malaria within the framework of a fixed effects regression model and included the independent variables of diesel fuels, pesticides, sanitation investments, income, and temperature in the analysis [68]. The third study investigated the consequences of plastic waste by developing a framework that analyzed human well-being based on environmental quality and economic security [136]. That study was performed at a regional level in Ghana in an urban and/or peri-urban area. The results of these three studies highlight the critical role of environmental factors on human and animal health and emphasize the need to develop comprehensive strategies to minimize the impact of air pollution, pollution, and plastic litter on human health and improve environmental quality.

## 4 Discussion

The results of this comprehensive review analysis of 108 articles show the diversity of economic approaches, research areas, and geographical contexts used in One Health research. As the search strategy focused on articles explicitly referring to One Health, relevant studies that apply the core principles of the concept but do not refer to it by name may have been overlooked. Other articles in languages other than English and German may also be relevant. A further limitation, although this affected only a minority of articles, is that potentially relevant studies were excluded if full-text access was not available. However, this exclusion was methodologically necessary, as a thorough analysis of the economic methods and regional classification of the studies required unrestricted access to methodological details. Without full-text access, a reliable evaluation of this information would not have been possible.

The studies were categorized by topic, but could have been categorized under more than one topic due to the interdisciplinary nature of the One Health approach. Issues in the areas of AMR, food safety or animal health influence each other and are interdependent. For example, the health of livestock, wildlife and pets can affect human health in terms of nutrition, psychology, and physique. Conversely, human behavior, such as our ecological footprint, can have a positive or negative impact on the environment and its biodiversity.

Linking geographical context and economic approaches was difficult for several reasons. Firstly, some One Health topics, such as environment, governance, or animal health, were severely under-represented, with a maximum of seven studies per category. About half of the studies did not classify areas as urban or rural. In addition, a large number of approaches were used for the economic approaches, making it difficult to compare them by topic and geographical context. Future research would need to focus on an economic analysis and then analyze the geographical context to conclude the corresponding distributions. In addition, most studies did not provide a rationale for their choice of economic methodology, making it difficult to evaluate the approach.

Despite the mixed economic approach, certain frequencies were identified depending on the main topic, such as the majority of mixed-method designs or CEAs for zoonoses. Mixed-method designs make it possible to visualize complex interrelationships, as is often the case in One Health. In addition to the effectiveness of interventions, their acceptability can also be assessed. CEAs can be used to prioritize interventions by weighing different interventions against each other. In this way, cross-sectoral investments can be assessed for their added value for public health. The use of rankings or SWOT analyses in the field of governance is also understandable, as they contribute to evidence-based decision-making and can help in action planning.

Across the articles, CBA dominated the analysis of costs (32 %), but often focused on single interventions, such as mass vaccination or disease surveillance, without considering cross-sectoral cost allocations or other influences. Although GDP and productivity loss measures were often used, few studies included environmental impacts or intangible costs such as animal welfare and plant health. Burdens measured, using DALYs and QALYs, adequately capture human health burdens, but neglect direct costs, such as the economic livelihoods of agricultural societies.

The studies found were characterized by a strong sectoral focus and a lack of cross-sectoral economic approaches. The GOHI represents progress in transdisciplinary collaboration, but its limited application highlights barriers to implementation. Limiting factors include the lack of publicly available data on which the GOHI is based, such as environmental indicators, or the lack of consideration of political and cultural frameworks. Logistic regression (11 %) identified risk factors such as vaccination costs, but systems approaches (e.g., causal loop analysis) to capture complex interspecies transmission pathways remained rare. Further economic search terms could be included for a follow-up article, such as ‘return on investment, added value, budget impact analysis, financial analysis/evaluation, monetary, investment, DALY, or QALY’ [11].

The geographical context of the One Health approaches tended to be neglected in the analysis. Studies focusing on the geographical context of African countries at the regional level (51 %) correctly addressed high-risk regions in the zoonotic and AMR area, but overlooked cross-border dynamics that require international cooperation. At 49 %, the rural studies focus on communities that are highly dependent on livestock farming. This approach is entirely justified. However, the lack of urban or peri-urban research (less than 14 %) neglects the risks posed by livestock markets in rapidly growing cities, among other things.

In economic models, it is essential to take regional realities into account. Mixed-method designs can be used for this purpose, for example, with the KPA approach, SWOT analysis, stochastic models, or rating in combination with the collection of burdens. In this context, it is essential to take greater account of the time factor. Many studies have focused on a short-term and quick solution. However, it should be noted that long-term, comprehensive preventive planning in the sense of ‘One Health’ would not only be more sustainable, but also more health-promoting.

Nevertheless, the geographical approach in the articles was comprehensible. For example, in the context of zoonoses (56 %), the place of investigation of the respective disease can be explained by the form of spread. The most common zoonosis in this article, rabies, was generally spread endemically by unvaccinated dogs and was correspondingly localized. The study distribution, which is mainly concentrated in West Africa (Liberia, Ivory Coast, Burkina Faso), Central Africa (Chad, Cameroon) and East Africa (Kenya, Tanzania), can be explained by the historical spread of this zoonosis in West and Central Africa, which was partly due to the trade routes during colonization to Europe. The persistent presence of diseases introduced several years ago highlights the long-term impact of human behavior on the environment. Awareness of the consequences of one's behavior must be understood in different time dimensions.

In addition to the endemic spread of zoonoses, the countries mentioned have a relatively low Human Development Index (approx. 0.39–0.6). A low Human Development Index can have direct consequences for the spread of diseases, such as a lack of prophylaxis (vaccinations), close human-animal contact, a lack of information about wound care, and political instability. A global comparison of the assessment of zoonoses using the Global One Health Index shows that the HDI is an indicator for the implementation of One Health measures [91]. Alternatively, alternative governance indicators such as the Worldwide Governance Indicators (WGI, World Bank) or the categorization of areas according to their status as emergent or endemic zones for certain zoonoses could be given greater consideration in the future [137].

The majority of AMR studies in developed European countries could be based on different overarching strategies. These include the European approach with EARS-Net (30 European countries), CAESAR (15 European countries), and the Action Plan (until 2027), which contribute to data collection. The coordinated collection and analysis of AMR data by European networks such as EARS-Net and CAESAR enables standardized surveillance in over 40 countries. These standardized data sources form the basis for comparative analyses and action plans. At the same time, there are differences between countries and pathogens, and some sectors, such as the environment or certain animal species, are less comprehensively covered. To effectively contain the spread of AMR and sustainably protect human and animal health, an integrated approach is crucial.

It is interesting to note that transnational measurement instruments for One Health were identified less frequently than local approaches. An international and global approach to health seems to correspond most closely to the holistic nature of the One Health concept. The ‘Global Dog Rabies Elimination Pathway’ (GDREP) tool [84] or the ‘Global Framework for Elimination of dog-mediated rabies’ (STOP-R-Index) [138], measuring instruments enable an international and global comparison of One Health approaches. They are the first examples of a geographically broader understanding of the One Health approach. It is positive to emphasize that human-to-dog ratios were recorded in both urban and rural areas. It should be noted that no geo-coordinates or GIS integrations are made. The STOP-R Index is characterized in particular by the consideration of national infrastructure indicators, such as the inclusion of natural risks like flooding. Fortunately, cultural factors such as the literacy rate and political stability are taken into account in the STOP-R Index. However, agricultural factors are neglected.

It is important to take cultural circumstances into account and to distribute the available resources fairly. This is an essential factor for the protection and support of small entrepreneurs, such as small farmers, and thus contributes to a healthy balance in the long term. In the long term, One Health for humans, animals, and the environment can only be achieved if decision-makers network at the regional level, thereby saving time and money. One measure could be a One Health region in which the health of humans, animals, and the environment is taken into account at regional level in future decisions in a region. In north-east Germany, for example, a project initiative to develop a One Health Region is being funded over nine years. The aim is to think about and establish health holistically at the regional level [139].

This analysis highlights the need to extend economic evaluations beyond anthropocentric calculations to adequately account for the complex value systems of the One Health philosophy. Future research should therefore focus on developing dynamic models that quantify the opportunity costs (cost of foregone benefits of the best alternative) of inaction in the human, animal, and environmental domains. Here, the geographical context can be taken into account for several variables.

## 5 Conclusion

This review emphasizes the need for a more nuanced understanding of economic approaches to One Health in geographical contexts. To fill this gap, it is recommended that future research should focus on developing and testing economic measures that can be applied in a geographical context. The focus should first be on a regional level, then on a national level, and finally on a multinational and global level. In addition, this review provides an overview of the different models developed. These can serve as a useful basis for the evaluation of One Health measures in other regional contexts. The variables analyzed are listed in the analysis. The variables are selected individually, taking into account the specific requirements and the regional context. This overview demonstrated that the implementation of the One Health concept generates measurable added value in various areas. There is a particular need for health economic evidence in the areas of the environment, digital solutions, and the prevention of other diseases (except zoonoses). In this context, the need for One Health regions that address One Health at a small-scale level and take into account the interdependence of human, animal, and environmental health becomes clear. The creation of these regions aims to promote healthy and sustainable development based on economic knowledge.

## CRediT authorship contribution statement

**Lena Schmeyers:** Writing – review & editing, Writing – original draft, Visualization, Validation, Project administration, Methodology, Investigation, Formal analysis, Data curation, Conceptualization. **Susan Thomschke:** Writing – review & editing, Validation, Project administration, Methodology, Investigation, Formal analysis, Data curation, Conceptualization. **Lena Victoria Mende:** Writing – review & editing, Validation, Formal analysis, Data curation. **Greet Stichel:** Writing – review & editing, Validation, Formal analysis, Data curation. **Daniel Schiller:** Writing – review & editing, Supervision, Resources, Project administration, Methodology, Investigation, Funding acquisition, Conceptualization. **Steffen Fleßa:** Writing – review & editing, Supervision, Resources, Project administration, Methodology, Investigation, Funding acquisition, Conceptualization.

## Funding sources

This work was supported by the German Federal Ministry of Research, Technology and Space (BMFTR) under grant number 03TR12L02 as part of the initiative T!Raum One Health-Region Vorpommern.

## Declaration of competing interest

The authors declare that they have no known competing financial interests or personal relationships that could have appeared to influence the work reported in this paper.

## Data Availability

Data will be made available on request.

## Appendix A Supplementary data

Supplementary material: Overview of Articles by Geographical and Economic Categories Image 1
